# Kidney failure risk equation and early renal risk in adults with sickle cell disease: comparison with KDIGO classification using measured GFR

**DOI:** 10.1080/0886022X.2026.2699576

**Published:** 2026-08-05

**Authors:** Yannick Mompango Engole, Jean-Robert Rissassy Makulo, Yannick Mayamba Nlandu, Aliocha Natuhoyila Nkodila, Justine Busanga Bukabau, Brady Makanzu, Yannick Mvita, Jean Michel Mavungu, François Musungayi Kajingulu, Vieux Momeme Mokoli, Augustin Luzayadio Longo, Marie-France Ingole Mboliasa, Evariste Mukendi Kadima, Chantal Vuvu Zinga, Clarisse Nsenga Nkondi, Jonathan Musa, Cedric Kabemba Ilunga, Raggue Mvibudulu, Blondy Mvete Mansoni, Beudin Lukoki, Nazaire Mangani Nseka, Ernest Kiswaya Sumaili

**Affiliations:** a Nephrology Unit, Kinshasa University Hospital, University of Kinshasa, Democratic Republic of the Congo; b Specialized Clinics of the Kinshasa, Democratic Republic of the Congo; c SANRU, Kinshasa, Democratic Republic of the Congo; d Cardiology Unit, Kinshasa University Hospital, Kinshasa, University of Kinshasa, Kinshasa, XI, Democratic Republic of the Congo

**Keywords:** Sickle cell disease, renal risk, measured glomerular filtration rate, albuminuria, kidney failure risk equation

## Abstract

Chronic kidney disease is a major complication of sickle cell disease (SCD), but early kidney injury frequently occurs despite preserved or increased glomerular filtration rate (GFR), potentially limiting the usefulness of conventional kidney failure prediction tools. We evaluated whether the Kidney Failure Risk Equation (KFRE) adequately reflects renal vulnerability in adults with SCD. In this multicenter cross-sectional study conducted in Kinshasa, Democratic Republic of the Congo, 241 adults with stable SCD underwent iohexol-measured GFR (mGFR) and albuminuria assessment. Renal risk was classified according to KDIGO criteria. The KFRE was calculated overall and in participants with CKD stage ≥3. Multivariable logistic regression identified factors independently associated with moderate-to-high renal risk. Overall, 35.7% of participants had moderate-to-high KDIGO renal risk. Despite this burden of renal risk, KFRE estimates remained very low overall and increased only in participants with CKD stage ≥3 (median risk: 5.98% at 2 years and 10.14% at 5 years). Increased renal risk was independently associated with vaso-occlusive crises (aOR 2.80, *p* = 0.012), leg ulcers (aOR 1.90, *p* = 0.030), stroke (aOR 2.66, *p* = 0.035), elevated LDH >220 U/L (aOR 3.19, *p* = 0.006), CRP ≥6 mg/L (aOR 3.04, *p* = 0.024), AST >40 IU/L (aOR 2.60, *p* = 0.002), and tricuspid regurgitant velocity ≥2.5 m/s (aOR 1.81, *p* = 0.041). More than one-third of adults with SCD had moderate-to-high renal risk despite preserved GFR. The discordance between KDIGO renal risk and KFRE estimates suggests that KFRE may underestimate early renal vulnerability in SCD. Measured GFR combined with albuminuria may improve early renal risk stratification and warrants prospective validation.

## PLAIN LANGUAGE SUMMARY

People with sickle cell disease are at high risk of kidney damage, but early kidney injury often develops before standard kidney function tests become abnormal. This makes it difficult to identify patients who are truly at risk using conventional prediction tools.

In this study, we evaluated kidney risk in 241 adults with sickle cell disease in Kinshasa, Democratic Republic of the Congo. We compared the Kidney Failure Risk Equation (KFRE), a widely used tool to predict kidney failure, with KDIGO renal risk classification based on a highly accurate measurement of kidney function (iohexol-measured glomerular filtration rate) and albuminuria.

More than one-third of participants had moderate-to-high renal risk according to KDIGO criteria, although most still had preserved kidney filtration. In contrast, KFRE predicted a very low risk of kidney failure for almost all participants, suggesting that it may not adequately identify early kidney vulnerability in sickle cell disease.

These findings indicate that relying on KFRE alone could miss patients with early kidney damage. Combining measured kidney function with albuminuria may provide a more accurate assessment of renal risk and should be evaluated further in prospective studies.

## KEY MESSAGES

What is known?

Kidney injury in sickle cell disease often precedes GFR decline, limiting conventional risk prediction tools.

What does this study add?

More than one-third of adults had moderate-to-high KDIGO renal risk despite preserved GFR and low KFRE scores.

What is the potential impact?

KFRE may underestimate early renal vulnerability; measured GFR plus albuminuria may improve risk stratification.

## Introduction

Sickle cell disease (SCD) is the most common monogenic hemoglobinopathy worldwide, affecting approximately 300,000 newborns annually, with the highest burden in sub-Saharan Africa, India, the Middle East, and populations of African ancestry in Europe and the Americas [1,2]. Caused by a point mutation in the β-globin gene leading to hemoglobin S (HbS) production, SCD is characterized by recurrent vaso-occlusion, chronic hemolysis, and endothelial dysfunction, resulting in progressive multisystem organ damage [3]. In addition to erythrocyte sickling, circulating microparticles and dysregulation of the protein C pathway amplify thrombo-inflammation and vascular injury, contributing to organ complications [4–6]. Among affected organs, the kidney is particularly vulnerable, and renal involvement is a major determinant of morbidity and mortality in adults with SCD [7,8].

Sickle cell nephropathy is a complex glomerulo-tubular disorder driven by chronic medullary hypoxia, glomerular hyperfiltration, ischemia-reperfusion injury, oxidative stress, and inflammation [9,10]. These mechanisms promote albuminuria, tubular dysfunction, and progressive chronic kidney disease (CKD), which may ultimately progress to kidney failure [11–15]. Early identification of renal vulnerability is therefore essential to improve surveillance, guide renoprotective interventions, and optimize enrollment and outcome assessment in clinical trials.

Current guidelines recommend assessment of albuminuria and glomerular filtration rate (GFR) for CKD evaluation [16,17]. In the general CKD population, the Kidney Failure Risk Equation (KFRE) accurately predicts progression to kidney failure [18,19]. However, its applicability in SCD remains uncertain because kidney injury often develops while GFR is still preserved or increased. Glomerular hyperfiltration may mask early nephropathy, whereas reduced muscle mass and enhanced tubular creatinine secretion lower serum creatinine concentrations, limiting the accuracy of creatinine-based estimated GFR [20–23].

We therefore evaluated whether the Kidney Failure Risk Equation adequately reflects renal vulnerability in adults with SCD by comparing its predictions with KDIGO renal risk stratification based on iohexol-measured GFR and albuminuria, while identifying the clinical and biological factors independently associated with increased renal risk in a high-burden sub-Saharan African population.

## Methods

### Study design and participants

This multicenter cross-sectional study was conducted between March 2023 and April 2024 among adults with stable sickle cell disease (SCD) in Kinshasa, Democratic Republic of Congo (DRC). Participants were recruited in collaboration with their primary healthcare providers across 14 centers, including the Center For mixed medical for anemia SS, Makala General Referral Hospital, Bokoko Medical Center, Akram Hospital, Center Messie, Clinique Élisabeth, Fondation Amza, Rezodrepa, Codek, Colombe, Kalaki Foundation, and Lisungi.

No formal sample size calculation was performed because this was a multicenter cross-sectional study using consecutive, non-probability convenience sampling. All eligible adults with stable sickle cell disease attending the participating centers during the study period were invited to participate. Exclusion criteria included conditions or treatments likely to affect renal function, such as hypertension (excluded because the parent study aimed to characterize early renal abnormalities in clinically stable SCD patients without major comorbidities known to independently affect kidney function), diabetes, obesity, urinary tract infection, menstruation, pregnancy, recent vaso-occlusive crisis, fever, acute chest syndrome, or recent (<1 month) exposure to non-steroidal anti-inflammatory drugs or agents interfering with creatinine secretion.

The study was approved by the Ethics Committee of the Faculty of Public Health, University of Kinshasa (Approval No. ESP/CE/143/2022). Written informed consent was obtained from all participants prior to inclusion.

### Study endpoints

The primary endpoint was the comparison between KDIGO renal risk stratification based on iohexol-measured glomerular filtration rate (mGFR) and albuminuria, and KFRE estimates of kidney failure risk.

Secondary endpoints included the prevalence of moderate-to-high KDIGO renal risk and the identification of clinical and biological factors independently associated with moderate-to-high renal risk.

### Clinical and laboratory assessments

Demographic and clinical data, including age, sex, body weight, height, and body mass index (BMI), were recorded. The mGFR was determined by plasma iohexol clearance (Omnipaque 350 mg/mL; GE Healthcare, Belgium). After intravenous injection of 3.45 mL iohexol, 5 mL blood samples were collected at 120, 180, 240, and 300 min from the contralateral arm. After centrifugation, samples were stored at −80 °C and shipped to the University of Liège (Belgium) for analysis. Plasma iohexol concentrations were measured by liquid chromatography–tandem mass spectrometry (LC-MS/MS) in an ISO 15189-accredited laboratory participating in external quality assessment (Equalis AB, Sweden) [24,25]. mGFR was calculated using the slope-intercept method and corrected with Brøchner–Mortensen Equation [26,27] and normalized for body surface area (BSA) which was calculated according to Dubois and Dubois Equation [28].

Urine samples were collected from the first morning void. The urinary albumin-to-creatinine ratio (UACR) was calculated as albumin (mg/L) divided by creatinine (mg/L), both determined by immunoturbidimetry (Cobas, Roche Diagnostics, Germany).

- UACR categories were defined as follows:
- Albuminuria grade A1: < 30 mg/g
- Albuminuria grade A2: 30–300 mg/g
- Albuminuria grade A3: > 300 mg/g [29].

The α1-microglobulin to creatinine ratio (A1M/Cr) was measured using the same analyzer and expressed in mg/g.

**Figure 1.**
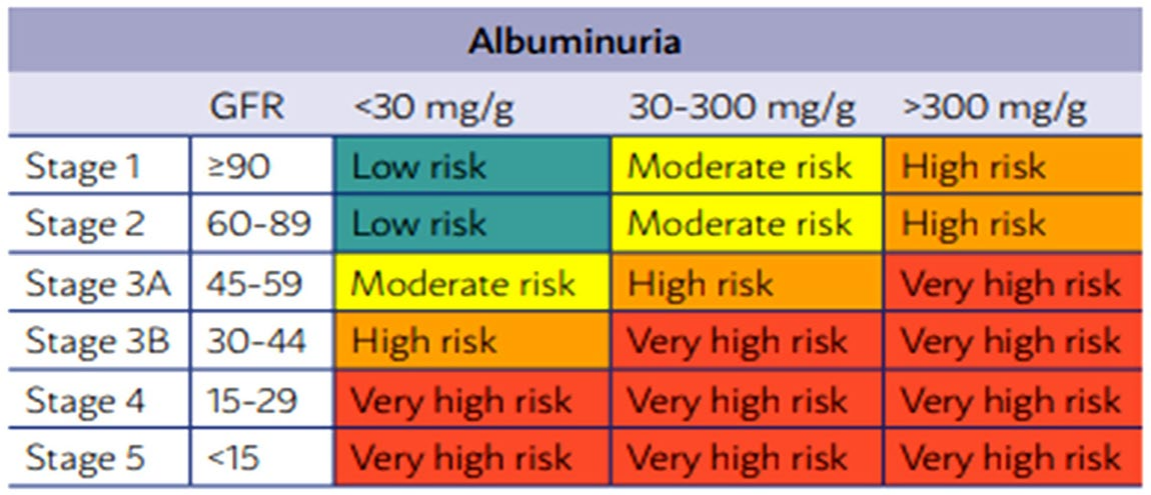
KDIGO classification of renal risk. KDIGO: kidney disease: improving global outcomes.

We evaluated the KDIGO renal risk using a combination of mGFR and albuminuria assessment. GFR was measured by iohexol plasma clearance, a reference method that provides accurate quantification of kidney filtration function. In addition, UACR was used to assess albuminuria, enabling detection of early glomerular damage. Patients were categorized into risk groups based on these renal parameters in accordance with established nephrology guidelines (Figure 1).

The KFRE was applied to estimate the risk of progression to kidney failure. The 4-variable KFRE model incorporates age, sex, estimated GFR, and urinary albumin-to-creatinine ratio to calculate the probability of kidney failure at 2 and 5 years. Estimated GFR for the KFRE calculation was derived using the CKD-EPI equation. The KFRE was not used to define renal risk categories but was applied to explore its performance in a population characterized by preserved or elevated measured GFR and early renal involvement [18].

Laboratory analyses included: Serum cystatin C, C-reactive protein (CRP), and ferritin measured by immunonephelometry (I-Chroma 2, Seoul, South Korea); Serum creatinine measured by the enzymatic method (Cobas, Roche Diagnostics, Germany); Complete blood count determined by impedance (Zybio, Seoul, South Korea); Lactate dehydrogenase (LDH), aspartate aminotransferase (AST), and bilirubin (total and direct) determined by kinetic methods on a semi-automated analyzer.

#### Operational definitions

- Low risk (green): mGFR (≥90-45 mL/min/1.73 m^2^) + Albuminuria grade A1
- Moderate increased risk (yellow): mGFR (≥90-45 mL/min/1.73 m^2^) + Albuminuria grade A2; Stage 3A + Albuminuria grade A1
- High risk (orange-red): mGFR ≥90-45 mL/min/1.73 m^2^ with severe albuminuria (UACR >300 mg/g); mGFR 45–59 mL/min/1.73 m^2^ with moderately increased albuminuria (UACR 30–300 mg/g); mGFR 15–44 mL/min/1.73 m^2^ regardless of albuminuria level; mGFR <15 mL/min/1.73 m^2^ regardless of albuminuria level [30].
- SCD with steady state: no history of vaso-occlusive crisis (VOC) or within one month of infection, hospitalization for acute chest syndrome, fever in the last month, current pregnancy [31].
- Recent vaso-occlusive crises: a history of vaso-occlusive crises during the previous three months [32].

A total of 241 patients with sickle cell disease (SCD) were consecutively enrolled in this study. Genotypic characterization was performed for all participants: 237 (98.3%) were homozygous for hemoglobin S (HbSS) and 4 (1.7%) had the HbSC genotype. These classifications were established based by cellulose acetate electrophoresis using the Gazelle device (Hemex Health, Portland, USA). Transthoracic Doppler echocardiography was performed by a single experienced cardiologist using a Mindray Consona 8 ultrasound system (Mindray, Shenzhen, China). During diastole, the left and right ventricular end-diastolic diameter were measured. Left ventricular mass (LVM) was calculated and indexed to body surface area (BSA). The left ventricular ejection fraction (LVEF) was determined using the modified biplane Simpson’s method, while the left atrial volume was also indexed to BSA. Tricuspid regurgitation velocity (TRV) was measured from multiple acoustic windows, and the highest velocity obtained was retained for analysis. Systemic vascular resistance (SVR) was considered low if < 700 dyns. s/cm^5^, calculated as: SVR = 80 × Mean Arterial Pressure/Cardiac Output [31]. Aortic stiffness was assessed by carotid-femoral pulse wave velocity (PWV), using the foot-to-foot method with the Popmeter device (Axelfil, France) [33].

### Statistical analysis

Data were entered into Microsoft Excel 2010, checked for consistency, and analyzed using the Statistical Package for Social Sciences (SPSS) version 26 (IBM Corp., Armonk, NY, USA). Continuous variables were expressed as mean ± standard deviation for normally distributed data and as median (interquartile range) for non-normally distributed data. Categorical variables were summarized as frequencies and percentages. Normality of continuous variables was assessed using the Shapiro–Wilk tests.

Comparisons between renal risk groups were performed using analysis of variance (ANOVA) for normally distributed variables or the Kruskal–Wallis test for non-normally distributed variables. Categorical variables were compared using the chi-square test or Fisher’s exact test, as appropriate. Multicollinearity was assessed using variance inflation factors (VIF), with values below 5 considered acceptable. Linearity of continuous predictors in the logit was assessed using the Box–Tidwell approach. No evidence of problematic multicollinearity or violation of linearity assumptions was observed.

The four-variable KFRE was calculated for all participants and summarized overall, across KDIGO renal risk categories, and among participants with CKD stage ≥3. Because of the cross-sectional design and the absence of longitudinal kidney outcomes, KFRE was evaluated descriptively as a complementary risk stratification tool rather than as a predictive outcome.

Factors associated with moderate to high renal risk were explored using logistic regression analyses. Variables with a p-value < 0.05 in univariate analysis and/or considered clinically relevant were entered into multivariate logistic regression models. Adjusted odds ratios (aORs) with 95% confidence intervals (CIs) were reported. Model calibration was evaluated using the Hosmer–Lemeshow goodness-of-fit test. Missing data were <5% for all variables; therefore, complete-case analysis was considered appropriate. A two-sided p-value < 0.05 was considered statistically significant.

## Results

Based on predefined criteria of renal risk, among the 241 included patients with sickle cell disease 155 patients (64.3%) were classified as low risk, 46 (19.1%) as moderate risk, and 40 (16.6%) as high renal risk. Thus, while the majority of patients were categorized as having a low probability of renal progression, more than one third of the cohort exhibited a moderate to high KDIGO renal risk profile.

The general characteristics of the study population stratified by renal risk category are summarized in Table 1. Sociodemographic variables did not differ significantly across the three groups. However, markers of disease severity were more prevalent in patients classified as high risk. A history of vaso-occlusive crises during the previous trimester was significantly more frequent in the high-risk group compared with the low- and moderate-risk groups [58 (37.4%) vs. 19 (41.3%) vs. 22 (55.0%); *p* = 0.033]. Similarly, leg ulcers were more commonly observed in patients at high renal risk [40 (25.8%) vs. 11 (23.9%) vs. 15 (37.5%); *p* = 0.035]. A history of stroke was also significantly more prevalent among patients classified as moderate and high renal risk [0 (0.0%) vs. 3 (6.5%) vs. 2 (5.1%); *p* = 0.007].

**Table 1.** Characteristics of population studied.

Variable	Overall (*n* = 241)	Low risk (*n* = 155)	Moderate risk (*n* = 46)	High risk (*n* = 40)	*p*
Sex					0.180
Male	99 (41.1)	63 (40.6)	15 (32.6)	21 (52.5)	
Female	142 (58.9)	92 (59.4)	31 (67.4)	19 (47.5)	
Folic acid use	223 (92.5)	142 (91.6)	44 (95.7)	37 (92.5)	0.722
Hydroxyurea use	88 (36.5)	55 (35.5)	16 (34.8)	17 (42.5)	0.701
Surgery	52 (21.6)	37 (23.9)	7 (15.2)	8 (20.0)	0.470
VOC in previous trimester	99 (41.1)	58 (37.4)	19 (41.3)	22 (55.0)	**0.033**
FH-SCD	137 (56.8)	83 (53.5)	29 (63.0)	25 (62.5)	0.393
History of blood Transfusion	219 (90.9)	137 (88.4)	44 (95.7)	38 (95.0)	0.199
Leg Ulcer	66 (27.4)	40 (25.8)	11 (23.9)	15 (37.5)	**0.035**
Priapism	17 (7.1)	10 (6.5)	2 (4.3)	5 (12.5)	0.306
Retinopathy	37 (15.4)	25 (16.1)	6 (13.0)	6 (15.0)	0.903
Aseptic necrosis of head femoral	40 (16.6)	28 (18.1)	8 (17.4)	4 (10.0)	0.503
Stroke	5 (2.1)	0 (0.0)	3 (6.5)	2 (5.1)	**0.007**
Tobacco	19 (7.9)	10 (6.5)	6 (13.0)	3 (7.5)	0.300
Alcohol	57 (23.7)	39 (25.2)	10 (21.7)	8 (20.0)	0.758

Data are expressed as mean ± standard deviation, absolute (n) and relative (in percent) frequencies. Abbreviations: FH-SCD: Familial history of Sickle Cell disease; VOC: Vaso-occlusive crisis.

Comparisons of hemodynamic, anthropometric, and echocardiographic parameters are presented in Table 2. No significant differences were observed across renal risk categories with respect to age, systolic and diastolic blood pressure, pulse pressure or anthropometric measures including body mass index and body surface area (all *p* > 0.05). Most echocardiographic parameters reflecting cardiac structure and systolic function were also comparable across groups. Elevated TRV (≥2.5 m/s) was significantly associated with renal risk category (*p* = 0.028). Patients in the moderate- and high-risk groups showed a higher prevalence of TRV ≥2.5 m/s (28.6% and 23.8%, respectively) compared with those in the low-risk group (11.8%).

**Table 2.** Hemodynamic and echocardiographic parameters in the population studied.

Variable	Overall (*n* = 241)	Low risk (*n* = 155)	Moderate risk (*n* = 46)	High risk (*n* = 40)	*p*
Age	25.9 ± 8.1	26.0 ± 7.8	25.3 ± 7.7	26.1 ± 9.8	0.862
SBP, mmHg	104.8 ± 12.3	104.9 ± 12.6	102.6 ± 10.4	107.0 ± 13.0	0.258
DBP, mmHg	59.0 ± 9.2	59.4 ± 9.5	57.4 ± 8.1	59.5 ± 9.2	0.417
PP, mmHg	45.8 ± 9.0	45.5 ± 9.0	45.2 ± 8.2	47.5 ± 9.9	0.407
Weight, Kg	49.9 ± 8.6	50.5 ± 8.0	49.5 ± 10.9	47.9 ± 7.6	0.228
Height, cm	159.9 ± 17.1	160.5 ± 15.6	157.8 ± 25.2	160.1 ± 9.5	0.641
BMI, Kg/m^2^	19.2 ± 3.0	19.4 ± 2.8	19.0 ± 3.7	18.8 ± 2.8	0.453
Fat mass,	20.1 (13.2–26.9)	19.9 (13.0–26.6)	22.9 (15.7–27.9)	14.6 (10.6–26.9)	0.245
PWV, m/sec	5.2 ± 1.2	5.1 ± 1.0	5.1 ± 1.0	5.2 ± 1.7	0.947
ABI,	1.1 ± 0.1	1.1 ± 0.1	1.1 ± 0.1	1.1 ± 0.1	0.685
LVEF, %	67.9 ± 10.0	67.4 ± 10.5	65.9 ± 8.2	71.2 ± 9.3	0.225
LVEDD, mm	50.5 ± 5.3	50.6 ± 5.5	51.9 ± 4.7	49.0 ± 5.2	0.262
RVEDD, mm	28.0 ± 4.6	27.7 ± 4.8	30.0 ± 3.4	27.3 ± 4.7	0.148
iLVM, g/m^2^	112.7 ± 26.7	114.2 ± 28.4	105.7 ± 16.0	114.4 ± 29.5	0.494
IVC, mm	12.6 ± 3.1	12.0 ± 3.2	13.3 ± 3.1	13.8 ± 2.4	0.066
TAPSE, cm	2.8 ± 0.4	2.8 ± 0.4	2.6 ± 0.5	2.6 ± 0.5	0.153
Cardiac output, L/min	6.8 ± 1.8	6.8 ± 2.0	7.1 ± 1.7	6.4 ± 1.5	0.560
TRV, m/sec	1.67 (1.45–1.84)	1.64 (1.43–1.80)	1.82 (1.66–2.16)	1.62 (1.38–1.94)	0.050
TRV cat					**0.028**
<2.5	106 (83.5)	75 (88.2)	15 (71.4)	16 (76.2)	
≥2.5	21 (16.5)	10 (11.8)	6 (28.6)	5 (23.8)	
SVR, dyn.s/cm^5^	950.0 ± 343.2	980.3 ± 393.0	963.2 ± 272.9	847.5 ± 180.6	0.483

Data are expressed as mean ± standard deviation, absolute (n) frequencies. Abbreviations: ABI: Ankle brachial index; BMI: Body mass index; BSA: Body surface area; DBP: Diastolic blood pressure; HR: Heart rate; IVC: Inferior venous cave; IVS: Interventricular septum; LVEDD: Left ventricular end diastolic diameter; LVEF: Left ventricular ejection fraction; iLVM: Indexed left ventricular mass; PP: Pulse pressure; PW: Posterior wall; PWV: Pulse wave velocity; RVEDD: Right ventricular end diastolic diameter; TAPSE: Tricuspid annular plane systolic excursion; TRV: Tricuspid regurgitation jet velocity; SBP: Systolic blood pressure; SVR: Systemic vascular resistance.

Biochemical and renal parameters according to renal risk category are detailed in Table 3. Markers of tubular and glomerular renal involvement increased progressively with renal risk level. Urinary A1M concentrations rose significantly from the low- to high-risk groups [13.5 (6.3–32.5) vs. 27.1 (16.4–58.4) vs. 63.6 (28.2–94.5); *p* < 0.001], as did the A1M/Cr [14.7 (7.3–32.6) vs. 35.3 (16.9–78.6) vs. 63.8 (28.1–103.8); *p* < 0.001]. Similarly, urinary albumin and UACR increased markedly across renal risk categories [5.3 (3.0–11.3) vs. 68.8 (35.8–106.0) vs. 475.0 (104.8–945.5); *p* < 0.001] and [6.6 (3.4–12.1) vs. 79.1 (44.2–129.7) vs. 601.1 (215.9–977.5); *p* < 0.001], respectively. In contrast, serum creatinine, cystatin C, mGFR, and estimated GFR using the CKD-EPI equation did not differ significantly between groups (all *p* > 0.05), indicating preserved global renal filtration across renal risk categories. Total blood counts were largely similar across groups, including hemoglobin concentration, hematocrit, red blood cell indices, and reticulocyte count (all *p* > 0.05). Platelet count differed modestly between groups, with higher values observed in the moderate-risk group (*p* = 0.039). Markers of hemolysis and inflammation increased progressively with renal risk severity. AST and LDH levels were significantly higher in the high-risk group [AST: *p* = 0.024; LDH: *p* = 0.023]. CRP concentrations also increased across renal risk categories [7.7 (2.5–12.7) vs. 8.1 (3.3–18.1) vs. 9.5 (4.3–24.5); *p* = 0.030].

**Table 3.** Biological parameters of population studied.

Variable	Over all (*n* = 241)	Low risk (*n* = 155)	Moderate risk (*n* = 46)	High risk (*n* = 40)	*p*
Urinary A1M, mg/L	20.6 (7.6–42.5)	13.5 (6.3–32.5)	27.1 (16.4–58.4)	63.6 (28.2–94.5)	**<0.001**
A1M/Cr, mg/g	19.6 (9.8–44.9)	14.7 (7.3–32.6)	35.3 (16.9–78.6)	63.8 (28.1–103.8)	**<0.001**
UACR, mg/g	11.0 (4.1–43.6)	6.6 (3.4–12.1)	79.1 (44.2–129.7)	601.1 (215.9–977.5)	**<0.001**
Creatinine, mg/dL	0.6 (0.5–0.7)	0.6 (0.5–0.7)	0.6 (0.5–0.7)	0.6 (0.5–0.9)	0.175
Cystatine C, mg/L	0.9 (0.8–1.0)	0.9 (0.7–0.9)	0.9 (0.8–1.1)	0.9 (0.7–1.1)	0.184
mGFR, mL/min/1,73 m^2^	126.0 (102.0–147.5)	124.0 (105.0–148.0)	126.5 (98.0–152.0)	127.5 (90.5–145.5)	0.802
eGFR crea, mL/min/1.73 m²	131.8 (130.1–134.2)	132.5 (130.3–135.1)	131.6 (126.8–136.0)	130.4 (125.6–135.2)	0.530
eGFR cys, mL/min/1.73 m²	104.1 (98.5–108.5)	105.2 (99.7–112.3)	97.8 (88.9–112.1)	102.9 (85.3–114.5)	0.227
eGFR crea + cys, mL/min/1.73 m²	118.4 (115.1–122.4)	118.9 (115.7–124.9)	114.1 (107.0–126.9)	115.4 (105.2–126.2)	0.343
Urea, mmol/L	4.8 (3.4–5.9)	4.9 (3.6–6.1)	4.8 (3.3–5.9)	4.5 (3.4–5.4)	0.239
Hb, g/dL	7.4 ± 1.7	7.4 ± 1.7	7.7 ± 1.5	7.2 ± 1.8	0.480
Hct, %	23.3 ± 5.5	23.3 ± 5.5	23.8 ± 5.0	22.6 ± 5.9	0.580
RBC, x 10^6^ /mm^3^	2.7 ± 0.7	2.7 ± 0.7	2.7 ± 0.6	2.6 ± 0.7	0.762
Reticulocyte count, %	16.5 ± 1.9	16.6 ± 1.9	16.0 ± 2.0	16.6 ± 2.0	0.144
WBC, x 10^3^/mm^3^	10.6 (8.7–14.0)	10.6 (8.9–14.1)	10.2 (8.3–13.1)	11.4 (8.3–16.4)	0.420
Platelets count, x 10^3^/mm^3^	156.2 (150.4–162.4)	156.2 (150.4–162.4)	158.2 (151.3–166.2)	152.2 (150.1–160.0)	**0.039**
AST, IU/L	33.7 (27.1–46.4)	33.2 (27.1–44.8)	33.8 (26.6–48.5)	40.5 (28.1–54.8)	**0.024**
Total Bilirubin, mg/dL	1.0 (0.9–1.2)	1.0 (0.9–1.2)	1.0 (0.9–1.3)	1.0 (0.9–1.2)	0.964
Direct Bilirubin, mg/dL	0.3 (0.2–0.5)	0.3 (0.2–0.5)	0.3 (0.2–0.7)	0.3 (0.3–0.5)	0.902
LDH, IU/L	210.6 (193.6–264.9)	212.0 (196.1–265.0)	200.2 (183.3–237.4)	220.8 (196.7–397.1)	**0.023**
Ferritin, ng/mL	236.3 (87.2–609.9)	224.6 (80.2–589.6)	380.8 (95.8–846.2)	191.8 (84.7–462.2)	0.217
CRP, mg/L	8.2 (3.3–18.1)	7.7 (2.5–12.7)	8.1 (3.3–18.1)	9.5 (4.3–24.5)	**0.030**
Fetal Hb, %	9.0 (8.0–13.0)	9.0 (8.0–13.0)	10.0 (8.0–14.0)	9.0 (6.8–13.0)	0.469

Data are expressed as mean ± standard deviation, median (interquartile range), absolute (n) and relative (in per cent) frequencies. Abbreviations: A1M/creatininuria: Alpha-1 microglobulin-creatininuria; AST: Aspartate amino transferase; CRP: C-reactive protein; DBP: Diastolic blood pressure; GFR: Glomerular filtration rate; transferase; Hb: Hemoglobin; HbF: Hemoglobin fetal; Hct: Hematocrit; LDH: Lactate dehydrogenase; MCV: mean corpuscular volume; MCHC: mean globular hemoglobin concentration; MHC: mean hae moglobin contentration; RBC: Red blood cells; UACR: Urinary Albumin-creatinine ratio; Urinary A1M: Urinary Alpha-1 microglobulin; WBC: White blood cells.

**Figure 2.**
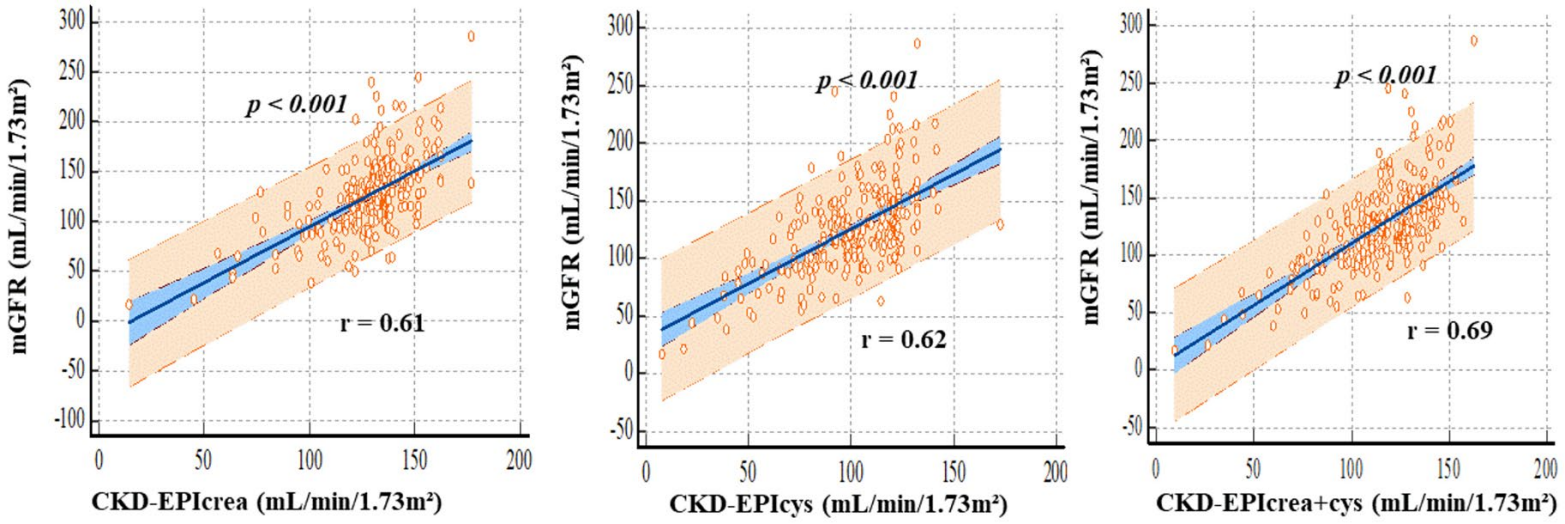
Correlation between measured GFR (mGFR) and estimated GFR. GFR: glomerular filtration rate; mGFR: measured glomerular filtration rate.

Figure 2 illustrates the relationship between mGFR and eGFR obtained using the CKD-EPI creatinine, CKD-EPI cystatin C, and CKD-EPI creatinine–cystatin C equations. All three equations showed significant positive correlations with mGFR (all *p* < 0.001). The strength of the correlation was moderate for CKD-EPIcrea (*r* = 0.61) and CKD-EPIcys (*r* = 0.62), and slightly stronger for the combined CKD-EPIcrea + cys equation (*r* = 0.69). Despite these significant associations, a substantial dispersion of observations around the regression lines was observed across the entire range of kidney function, particularly at higher GFR values.

The distribution of KFRE scores at 2 and 5 years in the overall cohort. KFRE values were highly concentrated near zero, with a markedly right-skewed distribution. No patient reached thresholds corresponding to intermediate or high risk of progression to kidney failure at either time point. The comparative distribution of KFRE scores at 2 and 5 years show slightly higher values at 5 years than at 2 years, while remaining within the low-risk range for all participants. Boxes represent the interquartile range (IQR), horizontal lines indicate the median, and whiskers represent the range. Median KFRE risk was 0.00011% (IQR 0.000004–0,001332) at 2 years and 0.00018% (IQR 0.00001–0.00211) at 5 years.

To align with the validated field of application of the KFRE, an additional analysis was performed among participants with CKD stage ≥3 (mGFR < 60 mL/min/1.73 m^2^). The distributions of KFRE scores at 2 and 5 years in this subgroup. In this CKD stage ≥3 subgroup, KFRE values remained predominantly in the low range, with a right-skewed distribution and slightly wider dispersion at 5 years compared with 2 years. KFRE-predicted risk of kidney failure at 2 and 5 years among patients with CKD stage ≥3. Median predicted risk was 5.98% (IQR 2.23–91.25) at 2 years and 10.14% (IQR 3.87–95.96) at 5 years.

After adjustment for potential confounders (Table 4), several clinical and biological variables remained independently associated with increased renal risk. A history of vaso-occlusive crises was associated with a nearly threefold increase in renal risk (adjusted OR [aOR] = 2.80; 95% CI: 1.88–3.73; *p* = 0.012). Leg ulcers (aOR = 1.90; 95% CI: 1.70–3.19; *p* = 0.030) and prior stroke (aOR = 2.66; 95% CI: 1.34–3.62; *p* = 0.035) also retained significant associations.

**Table 4.** Associated factors of high risk of CKD.

Variables	Univariate analysis		Multivariate analysis	
	p	OR (CI 95%)	p	aOR (CI 95%)
VOC in last trimester				
No		1		1
Yes	**0.002**	2.97 (1.99–3.90)	**0.012**	2.80 (1.88–3.73)
Leg ulcer				
No		1		1
Yes	**0.012**	2.77 (1.86–3.61)	**0.032**	2.03 (1.40–4.19)
Stroke				
No		1		1
Yes	**0.017**	3.55 (2.57–4.98)	**0.033**	2.96 (1.44–4.32)
Platelets count< 150,000/µL				
No		1		1
Yes	**0.032**	1.50 (1.34–3.24)	0.117	1.29 (0.33–1.62)
AST ≥ 40 IU/L				
No		1		1
Yes	**0.012**	2.94 (1.98–3.85)	**0.001**	3.10 (1.76–3.98)
LDH >220 IU/L				
No		1		1
Yes	**0.002**	3.43 (2.72–4.83)	**0.005**	3.09 (1.74–4.68)
CRP ≥ 6 mg/L				
No		1		1
Yes	**0.013**	3.09 (2.54–4.19)	**0.022**	3.14 (2.48–4.24)
TRV				
<2.5 m/s				
≥2.5 m/s	**0.033**	2.76 (1.56–5.48)	**0.041**	1.81 (1.19–3.22)

Abbreviations: AST: Aspartate aminotransferase; CRP: C-reactive protein; LDH: Lactate dehydrogenase; TRV: Tricuspid regurgitation jet velocity; VOC: vaso-occlusive crisis.

Among biological variables, elevated AST (aOR = 2.60; 95% CI: 1.76–3.38; *p* = 0.002), LDH > 220 U/L (aOR = 3.19; 95% CI: 2.57–4.48; *p* = 0.006), and CRP ≥ 6 mg/L (aOR = 3.04; 95% CI: 2.48–4.24; *p* = 0.024) were independently associated with renal risk. The echocardiography variable TRV ≥ 2.5 m/s (aOR = 1.81; 95% CI: 1.19–3.22; *p* = 0.041) was also associated with high renal risk. The association between thrombocytopenia and renal risk was no longer significant after multivariate adjustment (*p* = 0.617).

## Discussion

More than one-third of adults with sickle cell disease (SCD) in our cohort were classified as having moderate-to-high renal risk despite largely preserved kidney function. This finding is consistent with previous studies reporting a high burden of early kidney involvement in SCD, characterized by albuminuria and tubular injury preceding measurable decline in glomerular filtration rate (GFR) [10,14,34–39]. The relatively young age of our cohort may partly explain the low prevalence of advanced CKD and end-stage kidney disease, limiting extrapolation to older adults.

The principal finding of this study is the apparent discordance between KDIGO renal risk stratification and KFRE estimates. While KFRE predicted very low 2 and 5-year kidney failure risks in most participants, more than one-third already fulfilled criteria for moderate-to-high renal risk. This discrepancy is biologically plausible because early sickle cell nephropathy is characterized by glomerular hyperfiltration, preserved or increased GFR, and early glomerular and tubular injury rather than reduced filtration [20–22,34,36,38]. Since KFRE was developed and validated mainly in CKD stages 3–5 [18,19,40–43], it may not adequately reflect early renal vulnerability in SCD. Because of the cross-sectional design, our findings should not be interpreted as evidence of poor predictive performance but rather as indicating that KFRE may underestimate early renal risk in this population.

Higher renal risk was independently associated with vaso-occlusive crises, leg ulcers, stroke, and markers of hemolysis and inflammation, including LDH, AST, CRP, and elevated TRV. These findings support previous evidence linking disease severity, chronic hemolysis, endothelial dysfunction, systemic inflammation, and vasculopathy with progressive kidney injury [32,44–49]. Although platelet count has been associated with faster renal decline in longitudinal studies [50], it was not independently associated with renal risk after multivariable adjustment, suggesting that it mainly reflects overall disease activity rather than renal injury itself.

Our findings have important clinical implications. In SCD, reliance on estimated GFR or KFRE alone may overlook patients with substantial renal vulnerability. Combining iohexol-measured GFR with albuminuria and biomarkers of tubular injury provides a more comprehensive assessment of early kidney damage and may improve patient selection for closer surveillance, renoprotective interventions, and future clinical trials.

The main strengths of this study include the use of iohexol-measured GFR, a reference method that avoids the limitations of creatinine-based estimates in SCD, and the integrated evaluation of glomerular and tubular injury in a well-characterized African cohort. Limitations include the cross-sectional design, which precludes causal inference and assessment of KFRE predictive accuracy, the use of a single urine sample for albuminuria assessment, the relatively small number of participants with CKD stage ≥3, and the absence of longitudinal follow-up or genetic profiling. Prospective studies are needed to validate kidney-specific prediction models adapted to the unique pathophysiology of sickle cell nephropathy.

## Conclusion

More than one-third of adults with sickle cell disease had moderate-to-high KDIGO renal risk despite preserved glomerular filtration rate. Our findings suggest that the Kidney Failure Risk Equation may underestimate early renal vulnerability because kidney injury frequently precedes GFR decline. Until disease-specific prediction models become available, combining measured GFR with albuminuria may improve early renal risk stratification in adults with sickle cell disease.

## Data Availability

The data supporting the results of this study are available from the corresponding author, [Yannick Engole], upon reasonable request.

## E-mail and Orcid of Coauthors

- jrmakulo2016@gmail.com; https://orcid.org/0000-0001-5517-3281
- yannicknlandu@yahoo.fr; https://orcid.org/0000-0001-6158-0799
- nkodilaaliocha@gmail.com; https://orcid.org/0000-0002-1403-8451
- justinebuk@yahoo.fr; https://orcid.org/0000-0003-3567-6927
- bmaknzu@gmail.com; https://orcid.org/0000-0001-9167-3822
- m_yannick@live.fr; https://orcid.org/0009-0008-5268-3372
- mavungujeanmichel@gmail.com; https://orcid.org/0009-0000-3246-8984
- vieux.mokoli@gmail.com; https://orcid.org/0000.0002. 0536-24
- francoisKajingulu@gmail.com; https://orcid.org/0009-0007-9292-1315
- augustin.longo@gmail.com; https://orcid.org/0000-0001-5111-097X
- chantazinga2@gmail.com; https://orcid.org/0000-0001-5502-3814
- clarankondi@gmail.com; https://orcid.org/0009-0009-8991-5664
- jonathanmusa040@gmail.com; https://orcid.ord/0009-0009-5071-9244
- mboliasa@yahoo.fr; https://orcid.org/0000-0002-1936-7510
- evakad92@gmail.com; https://orcid.org/0009-0009-2545-3819
- c_ilunga2000@yahoo.fr; https://orcid.org/0009-0005-1057-8484
- raggue.mvibudulu@gmail.com; https://orcid.org/0009-0004-8566-0549
- blondymansoni@gmail.com; https://orcid.org/0009-0005-4623-6515
- beudinlukoki@gmail.com; https://orcid.org/0009-0001-3845-0643
- sumailiernest2015@gmail.com; https://orcid.org/0000-0003-1555-7197
